# Population levels of, and inequalities in, active travel: A national, cross-sectional study of adults in Scotland

**DOI:** 10.1016/j.pmedr.2017.09.008

**Published:** 2017-09-28

**Authors:** Jonathan R. Olsen, Richard Mitchell, Nanette Mutrie, Louise Foley, David Ogilvie, M74 study

**Affiliations:** a MRC/CSO Social and Public Health Sciences Unit, University of Glasgow, 200 Renfield Street, Glasgow G2 3QB, United Kingdom; b Centre for Research on Environment, Society and Health, Institute of Health and Wellbeing, University of Glasgow, 200 Renfield Street, Glasgow G2 3QB, United Kingdom; c Physical Activity for Health Research Centre, University of Edinburgh, St Leonard's Land, Holyrood Road, Edinburgh EH8 8A, United Kingdom; d MRC Epidemiology Unit & UKCRC Centre for Diet and Activity Research (CEDAR), School of Clinical Medicine, University of Cambridge, Box 285, Cambridge Biomedical Campus, Cambridge CB2 0QQ, United Kingdom

**Keywords:** Active travel, Physical activity, Urban areas, Rural areas, Health inequality

## Abstract

This study aimed to describe active travel (walking or cycling) in Scotland and explore potential demographic, geographic, and socio-economic inequalities in active travel. We extracted data for the period 2012–13 (39,585 journey stages) from the Scottish Household Survey. Survey travel diaries recorded all journeys made on the previous day by sampled individuals aged 16 + living within Scotland, and the stages within each journey. Descriptive statistics were calculated for journey stages, mode, purpose and distance. Logistic regression models were fitted to examine the relationship between the likelihood of a journey stage being active, age, sex, area deprivation and urban/rural classification. A quarter of all journey stages were walked or cycled (26%, n: 10,280/39,585); 96% of these were walked. Those living in the least deprived areas travelled a greater average distance per active journey stage than those in the most deprived. The likelihood of an active journey stage was higher for those living in the most deprived areas than for those in the least deprived (Odds Ratio (OR) 1.21, 95% CI 1.04–1.41) and for those in younger compared to older age groups (OR 0.44, 95% CI 0.34–0.58). In conclusion, socio-economic inequalities in active travel were identified, but – contrary to the trends for many health-beneficial behaviours – with a greater likelihood of active travel in more deprived areas. This indicates a potential contribution to protecting and improving health for those whose health status tends to be worse. Walking was the most common mode of active travel, and should be promoted as much as cycling.

## Highlights

• A quarter of all journey stages were actively travelled in Scotland, UK and 96% of those were walked.
• The proportion of journey stages made by active modes was greater for younger than older individuals.
• People living in the most deprived areas were more likely to report active travel than those in the least deprived areas.
• Walking remains by far the most likely mode of active travel and should be promoted as much as cycling.

## 1 Introduction

Physical inactivity has a major health effect worldwide (Lee et al., 2012) and the prevalence of non-communicable diseases and conditions linked to insufficient physical activity, such as coronary heart disease (Lee et al., 2012), has recently risen world-wide. Furthermore, those who are phsycially active are at lower risk of cardiovascular disease, then those who aren't (Celis-Morales et al., 2017). The World Health Organisation (WHO) is currently implementing the first European strategy on physical activity (Rütten et al., 2016), which aims to promote physical activity, reduce sedentary behaviours, remove environmental barriers to activity and provide equal opportunities to be active (World Health Organisation, 2015).

Individual-level demographic and socio-economic characteristics are important predictors of physical activity (Turrell et al., 2010). In addition, environmental settings can affect behaviours and restrict people from acting in a healthy way (Kamphuis and van Lenthe, 2013); for example, those living in rural settings often have lower levels of physical activity than those living in urban areas (Barrett et al., 2016, Hutchinson et al., 2014, Kurti et al., 2015, Patterson et al., 2014).

One strategy to increase physical activity is to shift journeys from motorised to ‘active’ modes of travel, such as making a journey on foot or by bicycle as opposed to by car. This strategy has been agreed globally as one of the seven best investments for increasing physical activity (Global Advocacy for Physical Activity, 2011). However, increasing active travel is likely to be easier in some settings, and for certain individuals, than others. It may be particularly challenging if, for example, rural residents have to travel long distances between home and their place of work or study (Hansen et al., 2015). The relationship between walking, cycling and socioeconomic status is complex (Shortt et al., 2014); studies have shown that individuals living in the most deprived areas of urban Australia were more likely to walk for transport than those living in the most affluent areas (Rachele et al., 2015), potentially offsetting the negative effects of other, less healthy behaviours for those living in disadvantaged areas (Turrell et al., 2013).

Scotland is a nation notorious for high levels of poor health and health inequality (Walsh et al., 2016). Yet in Scotland the prevalence of physical inactivity in adults, defined as not achieving at least 150 min of moderate or 75 min of vigorous intensity physical activity per week, is similar to that of other developed Western nations including the rest of the United Kingdom (U.K.) and the United States (U.S.) (Global Observatory for Physical Activity, 2016). With a large proportion of the Scottish population resident in an urban ‘central belt’ dominated by Glasgow in the west and Edinburgh in the east, the potential for active travel to make a significant contribution to increasing physical activity levels is clear and has been the subject of policy focus. Since 2011, adult active travel has been monitored annually using data from the Scottish Household Survey (SHS) at a national level through the Scottish Governments' National indicator in the ‘Active Scotland Outcomes Framework’ (Scottish Government, 2016). The indicator records the proportion of adults who made a walking journey of over 0.25 miles for a reason other than recreation during the previous week. At baseline (2011) 63% of Scottish adults aged 16 + achieved this target; this increased to 67% by 2014 (Brown et al., 2015).

Exploring levels of active travel remains important and further enquiry is required, particularly within Scotland, because many studies are either focused on one local area, use reported physical activity rather than transport for all routine journey stages taken during a single day, and/or have failed to examine active travel by socio-demographic or geographical subgroup. As highlighted in the previous sub-sections, it is important to understand prevalence in subgroups defined by age, sex, geography and socioeconomic status because of the possibility that interventions which are effective for some but not others will increase population level health inequalities. The aims of this study were, therefore, (a) to describe the proportion of journey stages actively travelled (walked or cycled) in Scotland by mode and purpose, (b) to explore differences in distance travelled by socio-economic factors, and (c) to explore demographic, geographic and socio-economic factors as correlates of the likelihood of active travel.

## 2 Methods

### 2.1 Survey data

Travel diary data were obtained from the 2012–13 Scottish Household Survey (SHS), a nationally representative rolling cross-sectional survey of adults aged 16 + selected from a cluster-random sample of households in Scotland (Scottish Goverment, 2015). The SHS is a general purpose survey covering a wide range of issues, including transport and travel. Face-to-face interviews were conducted and participants completed a travel diary which detailed all journeys undertaken the previous day. Each diary was divided into individual journey stages which describe each phase of a journey (e.g. one journey may include three stages: walk to bus stop, travel on bus, and walk to destination). For each journey stage, data collected included the origin, destination, purpose (assigned to all stages that comprised a given journey), distance, and mode of travel. Journey stage distances were calculated by Transport Scotland using the straight-line distance between origin and destination (Transport Scotland, 2010).

The response rate of the 2012 Scottish Household Survey was 67.2%. The rate varied by region, whereby the lowest rates were in urban/city regions (three lowest: Aberdeen City (57%), Midlothian (60%) and Glasgow City (59%)) and the highest were in rural areas (three highest: Orkney Islands (86%), Shetland Islands (78%), and North Ayrshire (78%)) (Scottish Goverment, 2014). After weighting, 48.3% (n: 19,112) of the sample were male, similar to the 2013 mid-year estimate for the population aged 16 and over in Scotland (48.0%, n: 2,120,629) (National Records of Scotland, 2016). The individual-level weighting corrected for differences in selection probabilities across areas of Scotland and socioeconomic status, allowing comparisons in active travel between these groups.

Travel diary data were provided for the 2012–13 survey for all areas of Scotland. Each participant's home, as well as the origin and destination of each journey stage, was assigned to a Scottish Intermediate Zone; these zones are geographical polygons containing groups of approximately 4000 household residents which respect physical boundaries and natural communities, have a regular shape and contain households with similar social characteristics (Scottish Government, 2011).

The origin of each journey stage was linked to: the eight-category Scottish urban/rural index (which classifies the urbanicity of the area (Scottish Government, 2014)); a local authority (Intermediate Zones follow local authority boundaries); and quintile of the 2012 Scottish Index of Multiple Deprivation (SIMD), a multivariate, area-based indicator of relative social, economic and environmental deprivation (Scottish Government, 2012) (1 = most deprived, 5 = least deprived). For this study, an area was classified as ‘urban’ if it was within either of the following two Scottish urban/rural index categories; (1) Large Urban Areas: settlements of over 125,000 people, or (2) Other Urban Areas: settlements of 10,000 to 125,000 people (Scottish Government, 2014).

### 2.2 Active travel

Active travel was defined as a journey stage that was either walked or cycled; these are the only active modes of travel captured in the survey. All journey stages were included and ‘active travel’ was an attribute assigned to each journey stage.

### 2.3 Statistical analysis

#### 2.3.1 Description of journey stages

##### 2.3.1.1 Descriptive statistics

Summary statistics described population characteristics in terms of sex, age, urban/rural classification, and area socio-economic status (SIMD quintile). The proportion of journey stages made by an active mode of travel was described for each stratum. As geographical location was provided for both the respondent's home and the origin of each journey stage, descriptive statistics were calculated for both home and stage-origin deprivation quintile. All analyses were conducted using both classifications, but there were no substantive differences in the results. We therefore present only the results based on deprivation quintile of residence.

##### 2.3.1.2 Journey mode and distance travelled

Mode of travel was described as a proportion of total travel for all journey stages and individually by socio-economic status (SIMD quintile). The distribution of journey purpose was described for all journeys, active journeys only and by socio-economic status (SIMD quintile).

Linear regression was used to estimate coefficients (β) for the continuous variable of distance (km) of active journey stages by deprivation quintile, to represent the mean change in journey distance for each increment of deprivation quintile.

#### 2.3.2 Likelihood of an active journey stage

##### 2.3.2.1 Multivariable models

Journey stage was the unit of analysis, and we assessed the likelihood of a stage being ‘active’ by regressing the binary outcome variable for each stage (active yes/no) against the explanatory variables in a logistic model. Models were firstly fitted without covariates and then adjusted for age, sex, local authority, health status, rural urban status, deprivation and employment. The models were performed without adjustment for urban rural status and we found no substantial change in the results. Models took account of clustering of journey stages within individuals using the cluster option within STATA. The cluster command specified that observations might be clustered within individuals but would be independent between individuals.

Data were weighted to correct for differences in selection probabilities between areas of Scotland, the number of adults in different sized households, and days on which people were available for interview (Scottish Goverment, 2014). Models were estimated using both weighted and unweighted data; this made no substantial difference to the main outcomes of the analysis.

Analysis was performed in STATA/SE 14.0.

## 3 Results

### 3.1 Journey stage characteristics

A total of 39,585 journey stages were collected in SHS travel diaries during 2012–13. A greater proportion of the respondents were women than men (51.7% (n: 20,473)) and more lived in urban areas than in rural (69.2% (n: 27,385)) (Table 1).

**Table 1:** Number and proportion of active stages by individual and geographical characteristics (weighted).

	Active	%	Other	%
Sex				
Male	4945	25.88	14,167	74.12
Female	5334	26.06	15,139	73.94

Rural urban classification				
Rural	2937	24.07	9263	75.93
Urban	7343	26.81	20,042	73.19

Age band				
16 to 24	2071	37.03	3523	62.97
25 to 34	1884	29.40	4524	70.60
35 to 44	1745	24.12	5490	75.88
45 to 59	2202	20.81	8378	79.19
60 to 74	1747	23.45	5702	76.55
75 plus	631	27.21	1689	72.79

Residential deprivation quintile				
5 (least deprived)	2253	24.44	6964	75.56
4	1741	23.15	5780	76.85
3	2106	25.63	6112	74.37
2	2088	27.75	5435	72.25
1 (most deprived)	2092	29.44	5015	70.56
Total	10,280	25.97	29,305	74.03

Urban areas had a greater proportion of active journey stages than rural areas, as did the 16-to-24 age group compared to the other age groups. People living in the most deprived quintiles reported a greater proportion of active stages than those living in the least deprived areas.

### 3.2 Likelihood of an active journey stage

Those in older age groups were less likely to report an active journey stage than those in the 16-to-24 age group; this difference in likelihood was in the same direction across all age group comparisons to the 16-to-24 age group. Journey stages reported by those living in the most deprived areas were more likely to be actively travelled than those living in the least deprived areas (OR 1.21, 95% CI 1.04 to 1.41) (Table 2). There were no significant differences in the likelihood of an active journey stage being reported between those living in urban and rural areas when adjusted for local authority, sex, age, employment and health status. Sensitivity analysis was performed without adjustment for local authority and the results remained similar. We present the model that includes adjustment for this.

**Table 2:** Multivariable models showing likelihood of a journey being active by demographic, geographic and socio-economic factors (weighted).

	Unadjusted			Adjusted ~		
	OR	LL-UL 95% CI	p	OR	LL-UL 95% CI	p
Gender						
Male	REF					
Female	1.01	0.92, 1.10	0.91	0.96	0.88, 1.06	0.413

Rural urban classification						
Rural	REF					
Urban	1.15	1.02, 1.30	0.02	1.07	0.95, 1.21	0.275

Age						
16 to 24	REF					
25 to 34	0.70	0.59, 0.83	< 0.001	0.66	0.55, 0.79	< 0.001
35 to 44	0.56	0.47, 0.66	< 0.001	0.53	0.44, 0.63	< 0.001
45 to 59	0.46	0.39, 0.54	< 0.001	0.42	0.36, 0.50	< 0.001
60 to 74	0.54	0.46, 0.63	< 0.001	0.40	0.32, 0.49	< 0.001
75 plus	0.65	0.53, 0.79	< 0.001	0.44	0.34, 0.58	< 0.001

Residential deprivation quintile						
5 (least deprived)	REF					
4	0.93	0.80, 1.08	0.33	0.95	0.82, 1.11	0.53
3	1.02	0.88, 1.18	0.77	0.99	0.86, 1.15	0.91
2	1.25	1.07, 1.45	0.00	1.13	0.97, 1.31	0.12
1 (most deprived)	1.33	1.14, 1.54	< 0.001	1.21	1.04, 1.41	0.02

Note: Models were adjusted for all other variables in the table and for local authority, gender, age, employment, urbanicity, deprivation and health status.

### 3.3 Travel by mode, purpose and socio-economic status

The weighted numbers and proportions of the modes of travel used in all journey stages are described in Table 3. Most active journey stages were walked (24.9% of all journey stages (n:9837)) and those made by bicycle represented a much smaller proportion of all journey stages overall (1.1% (n:443)).

**Table 3:** Number and proportion of total journey stages by individual modes (weighted).

Mode of travel	Num	%
Active travel		
Walking	9837	24.9
Bicycle	443	1.1

Motorised travel		
Car/van as driver	19,447	49.1
Car/van as passenger	5190	13.1
Motorcycle/moped	35	0.1
Taxi/minicab	525	1.3

Public transport		
Ordinary (service) bus	3115	7.9
School bus	92	0.2
Works bus	81	0.2
Train	686	1.7
Underground	37	0.1

Other		
Other	98	0.2
Total	39,585	100

Motorised modes of transport contributed the largest share, and half of all journey stages were made by car or van as a driver (Table 3). Public transport contributed 10% of stages, and ordinary bus services were the largest mode within the public transport group (7.9% (n: 3115)).

Most journey stages were completed for the purpose of travelling to work or education as the ultimate destination (Table 4) and the stages of these journeys were less likely to be made by active modes of travel than those of journeys for all purposes apart from ‘Other’. The greatest proportion of active travel was observed for stages of journeys to reach destinations for the purpose of exercise (60.1%), followed by those made for the purpose of shopping or personal appointments (28.0%).

**Table 4:** Journey purpose by active or non-active travel (weighted).

Journey purpose	Active	%	Other	
Work/education	3769	21.5	13,770	78.5
Social/visiting friends or family	1671	21.8	5988	78.2
Shopping/appointment	2582	28.0	6645	72.0
Exercise	1995	60.1	1325	39.9
Other	263	14.3	1577	85.7
Total	10,280	26.0	29,305	74.0

Note: Row percentages show the proportion of active or non-active journey stages for each travel mode.

**Fig. 1:**
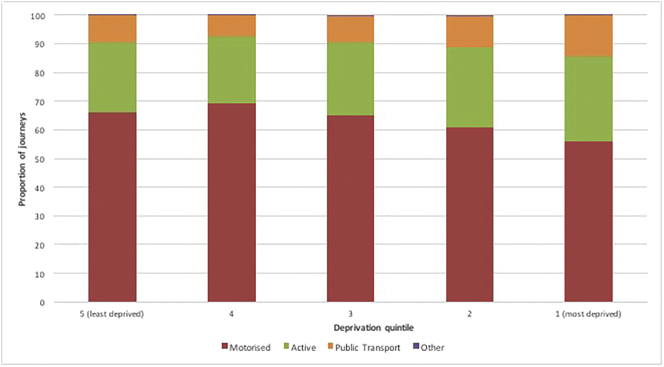
Journey stage mode by deprivation quintile (weighted).

For all deprivation quintiles the most frequently reported mode of transport was ‘motorised’ (excluding public transport) (Fig. 1). The proportion of motorised journey stages (excluding public transport) was highest in the two least deprived quintiles (4 and 5) and decreased slightly as deprivation increased. The frequency of active travel was highest in the most deprived quintile and decreased as areas became less deprived, apart from a slight increase in the least deprived quintile.

The average distance of an active journey stage was significantly less than that for a non-active stage (1.23 km vs. 10.38 km) (Table 5). Distance travelled also varied by respondents' deprivation quintile; for active journey stages, those living in the least deprived areas travelled a greater average distance than those in the most deprived areas.

**Table 5:** Mean distances of active and non-active journey stages by deprivation quintile (weighted).

Active journey distance (km)	Mean distance (km)	Coef	LL-UL 95% CI	p
All journeys				
Other	10.38	REF		
Active	1.23	− 9.16	− 9.53, − 8.78	< 0.0001

Residential deprivation quintile				
5 (least deprived)	1.34	REF		
4	1.25	− 0.09	− 0.24, 0.06	0.22
3	1.25	− 0.09	− 0.23, 0.05	0.20
2	1.11	− 0.23	− 0.37, − 0.09	0.00
1 (most deprived)	1.16	− 0.18	− 0.32, − 0.04	0.01

Note: Coefficients present the relationship of a one unit change from ‘non-active’ to ‘active’ travel on distance travelled compared to reference category.

## 4 Discussion

### 4.1 Summary

The aims of our study were to describe actively travelled journey stages in Scotland during 2012–13 by mode and purpose, to explore demographic, geographic, and socio-economic factors as correlates of active travel, and to investigate differences in distance travelled by socio-economic factors.

We found that the proportion of journey stages made by active modes was greater for younger than older individuals, and that most journey stages were completed for the purpose of travelling to work or education. People living in the most deprived areas were more likely to report an active journey stage than those in the least deprived areas, but the mean distance travelled for an active journey stage was less than for those in the least deprived areas. For a single active journey stage a small difference in average distance may seem unimportant, but in the course of a year over an entire population these modest differences could represent meaningful differences in physical activity. Journeys travelled for the purpose of travelling to or from an exercise activity had the greatest proportion of active stages for that purpose, which suggests that a majority of those travelling for exercise are gaining further health benefits by actively travelling to the exercise location.

Travelling as a driver or passenger in a car/van was the most frequent mode of travel. For active stages alone, the number of stages walked was much higher than the number cycled.

### 4.2 Comparison with other literature

We found that a quarter (26%) of journey stages across Scotland were made by active modes. This is similar to the 2012 UK National Travel Survey (NTS) and the Scottish NTS, in both of which 24% of journey stages were made by active modes (Department for Transport, 2013, Transport Scotland, 2014b).

Across Scotland, our results showed that those living in the most deprived areas have a greater likelihood of an active journey stage than people in less deprived areas. A similar pattern has been reported in previous studies (Heesch et al., 2015). A study examining the impact of socioeconomic and physical environment deprivation in UK adults (Richardson et al., 2010) found that regardless of environmental situation, those living in the lowest income groups had greater odds of active travel for non-recreational trips (Rind et al., 2015). A comprehensive European systematic review of physical activity found that many studies reported an association, but that these differed in direction; there were, however, fewer studies of active travel than of other physical activity domains (Beenackers et al., 2012). A more recent study of 40-to-65-year-old adults in urban Australia found that individuals living in the most deprived areas were more likely to walk and use public transport than those living in more affluent areas, but this relationship was not found for cycling (Rachele et al., 2015). Our national study including both rural and urban areas showed a similar pattern when walking and cycling were combined. Due to a low frequency of cycling, we were unable to distinguish between walking and cycling journey stages in our analyses.

Studies of older adults found that access to shops and facilities encouraged a higher number of trips for walked or cycled journeys, specifically if having several amenities within a five minute walk of home (Davis et al., 2011). A meta-analysis of travel and the built environment literature also found that walking was strongly related to land use diversity, intersection density and a greater number of destinations within walking distance (Ewing and Cervero, 2010). This could suggest why our study found a greater number of journey stages completed by those living in the most deprived areas but these were of shortest distances, previous Scottish studies found that the most deprived areas tended contained the highest number of services and shops (Macintyre et al., 2008).

Similarly to previous studies in the U.K. (Hutchinson et al., 2014), Australia (Heesch et al., 2015), North America (Waygood et al., 2015) and the Netherlands (Scheepers et al., 2013) we found that those living in urban areas were more likely to actively travel a journey stage than those living in rural areas. In addition, our results highlighted that, although there was a difference in active travel between urban/rural areas, when adjusted for age, sex, employment and socioeconomic status the difference was not significant.

Most journey stages in our sample were completed for travel to the workplace, which can be associated with high quantities of sedentary behaviour, especially for desk-based workers (Chu et al., 2016). Physical activity and active travel can be encouraged through investments in high-quality sustainable transport infrastructure for the journey to work (Panter et al., 2016), and/or by employers through active travel interventions including behaviour change programmes, workplace travel plans and financial incentives (Petrunoff et al., 2016). Although active travellers in urban areas may be exposed to additional health risks such as air pollution (Doorley et al., 2015), the modelled health benefits of active travel substantially outweigh any harm caused by pollution in most settings (Tainio et al., 2016).

While much attention in the U.K. and world-wide has focused on promoting cycling, cycling accounted for only 4% of all active journey stages, compared to walking which accounted for the remaining 96%. Walking is a familiar, convenient and free mode of transport and exercise (Audrey and Procter, 2015), for which infrastructure is well established in most urban and some rural areas. Since it remains by far the most likely mode of active travel in many countries, it should be promoted as much as cycling.

### 4.3 Strengths and limitations of the study

The SHS travel diaries are part of an on-going repeat cross sectional survey, using a large, randomly selected and representative population sample, and applying largely consistent methods and population weighting in each wave. This study design does not follow up the same participants over time, so individual behaviour changes cannot be extracted from the data. Due to changes in the wording of the question for the reporting of walked journeys from 2012 we were unable to conduct a repeat cross-sectional analysis to describe change in active travel over time.

The SHS collects information on two active modes of travel only; walking and cycling. Whilst some definitions of active travel do include public transport in recognition of the walking needed to access the motorised systems, our definition of active travel, which was restricted to walking and cycling, is in agreement with relevant international literature (Flint and Cummins, 2016, Jarrett et al., 2012). Since we identified active travel at the level of the stage rather than the journey, it is likely that we ascertained all ‘meaningful’ walking or cycling as part of longer public transport journeys and that this was included in analysis.

The SHS travel diary collects information for all stages of a journey undertaken between its ultimate origin and destination. This allowed us to analyse stage data and maximise the sample size for our analysis. The SHS calculates journey distances using the straight-line distance between two points. This is likely to under-report the true distance of journeys, for which actual routes follow the road network and are usually longer, particularly for those living in remote rural areas (Transport Scotland, 2014a). Meaning further exploration of distances travelled, such as total distance travelled by deprivation quintile and number of stages, may be unwise using this dataset.

## 5 Conclusions

A quarter of all journey stages in Scotland, UK were actively travelled, and 96% of those were walked. Walking therefore remains by far the most frequently used mode of active travel, and remains a more likely public health intervention since many more people walk than cycle, specifically for shorter walkable distances. Future studies should explore how socio-economic status is related to active travel in terms of purpose and distance of travel.

Differences in levels of active travel remain between socio-economic groups. Most health inequalities are largely unfavourable to the most deprived groups in the population, but in the case of active travel in Scotland they run in the opposite direction, in that those living in the most deprived areas are the most likely to report active travel. Despite this, it is important that active travel is promoted regardless of socio-economic status; partly because of important health outcomes for which physical activity reduces risk and which are not strongly socially patterned, and partly for environmental co-benefits including reducing fossil fuel consumption, reducing vehicle emissions, and the preservation or enhancement of infrastructure to support walking and cycling.

## Consent for publication/approvals

Informed consent was obtained from each participant prior to participation by the SHS interviewer for use of SHS travel diary data by the Scottish Government and other parties; who subsequently require Scottish Government approval. Approval for use of Scottish Household Survey (SHS) travel diary data for the purposes of this study was granted by the Scottish Government (Ref: A10776862).

## Funding

This study was funded by the NIHR Public Health Research programme (project number 11/3005/07: see http://www.nets.nihr.ac.uk/projects/phr/11300507). JO and RM are funded by the Medical Research Council (MRC) as part of the Neighbourhoods and Health Programme (MC_UU_12017/10). DO is funded by the MRC (MC_UU_12015/6). LF is funded by the Centre for Diet and Activity Research (CEDAR), a UKCRC Public Health Research Centre of Excellence. Funding from the British Heart Foundation, Cancer Research UK, Economic and Social Research Council, Medical Research Council, the National Institute for Health Research, and the Wellcome Trust, under the auspices of the UK Clinical Research Collaboration, is gratefully acknowledged. The views expressed are those of the authors and not necessarily those of the NHS, the NIHR or the Department of Health. This article presents independent research funded by the National Institute for Health Research (NIHR). The views and opinions expressed herein are those of the authors and do not necessarily reflect those of the NIHR PHR programme or the Department of Health. The researchers were independent of the funders; the funders had no role in study design, data collection and analysis, the decision to publish, or the preparation of the manuscript.

## Contributors

JO designed and conducted the analysis in collaboration with RM, NM and DO, and all authors contributed to the interpretation of analysis. JO prepared the first draft of the manuscript, with all authors contributing to its main content and revising it with critical comments. All authors have read and approved the manuscript prior to submission, and agree to be accountable for all aspects of the work in ensuring that questions related to the accuracy or integrity of any part of the work are appropriately investigated and resolved.

## Competing interests

RM is a board member of a charity which promotes walking and active travel, and receives no remuneration for this. The other authors declare no conflicts.

## Data sharing statement

For further information please refer to the MRC Epidemiology Unit data sharing portal at http://epi-meta.medschl.cam.ac.uk.
